# Supplementary material for: OpenMM 7: Rapid development of high performance algorithms for molecular dynamics
Source: PLoS Comput Biol. 2017 Jul 26;13(7):e1005659. doi: 10.1371/journal.pcbi.1005659 (PMC5549999; doi:10.1371/journal.pcbi.1005659)
Supplement: S1 Source Code — (ZIP) [file pcbi.1005659.s002.zip › OpenMM-7.0.1-Source/docs/api-c++/generated/OpenMM.AmoebaVdwForce.html]

AmoebaVdwForce — OpenMM @OPENMM\_MAJOR\_VERSION@.@OPENMM\_MINOR\_VERSION@ documentation


# AmoebaVdwForce¶

*class* `OpenMM::``AmoebaVdwForce`¶
:   This class implements a buffered 14-7 potential used to model van der Waals forces.

    To use it, create an `AmoebaVdwForce` object then call `addParticle()` once for each particle. After a particle has been added, you can modify its force field parameters by calling `setParticleParameters()`. This will have no effect on Contexts that already exist unless you call `updateParametersInContext()`.

    A unique feature of this class is that the interaction site for a particle does not need to be exactly at the particle’s location. Instead, it can be placed a fraction of the distance from that particle to another one. This is typically done for hydrogens to place the interaction site slightly closer to the parent atom. The fraction is known as the “reduction factor”, since it reduces the distance from the parent atom to the interaction site.

    Methods

    |  |  |
    | --- | --- |
    | `AmoebaVdwForce` | Create an Amoeba VdwForce. |
    | `getNumParticles` | Get the number of particles |
    | `setParticleParameters` | Set the force field parameters for a vdw particle. |
    | `getParticleParameters` | Get the force field parameters for a vdw particle. |
    | `addParticle` | Add the force field parameters for a vdw particle. |
    | `setSigmaCombiningRule` | Set sigma combining rule |
    | `getSigmaCombiningRule` | Get sigma combining rule |
    | `setEpsilonCombiningRule` | Set epsilon combining rule |
    | `getEpsilonCombiningRule` | Get epsilon combining rule |
    | `getUseDispersionCorrection` | Get whether to add a contribution to the energy that approximately represents the effect of VdW interactions beyond the cutoff distance. |
    | `setUseDispersionCorrection` | Set whether to add a contribution to the energy that approximately represents the effect of VdW interactions beyond the cutoff distance. |
    | `setParticleExclusions` | Set exclusions for specified particle |
    | `getParticleExclusions` | Get exclusions for specified particle |
    | `setCutoff` | Set the cutoff distance. |
    | `getCutoff` | Get the cutoff distance. |
    | `getNonbondedMethod` | Get the method used for handling long range nonbonded interactions. |
    | `setNonbondedMethod` | Set the method used for handling long range nonbonded interactions. |
    | `updateParametersInContext` | Update the per-particle parameters in a `Context` to match those stored in this `Force` object. |
    | `usesPeriodicBoundaryConditions` | Returns whether or not this force makes use of periodic boundary conditions. |

    Enum: NonbondedMethod

    |  |  |
    | --- | --- |
    | **NoCutoff** | No cutoff is applied to nonbonded interactions. The full set of N^2 interactions is computed exactly. This necessarily means that periodic boundary conditions cannot be used. This is the default. |
    | **CutoffPeriodic** | Periodic boundary conditions are used, so that each particle interacts only with the nearest periodic copy of each other particle. Interactions beyond the cutoff distance are ignored. |

    `AmoebaVdwForce`()¶
    :   Create an Amoeba VdwForce.

    int `getNumParticles`() *const*¶
    :   Get the number of particles

    void `setParticleParameters`(int *particleIndex*, int *parentIndex*, double *sigma*, double *epsilon*, double *reductionFactor*)¶
    :   Set the force field parameters for a vdw particle.

        |  |  |
        | --- | --- |
        | Parameters: |  |

        - **particleIndex** – the particle index
        - **parentIndex** – the index of the parent particle
        - **sigma** – vdw sigma
        - **epsilon** – vdw epsilon
        - **reductionFactor** – the fraction of the distance along the line from the parent particle to this particle at which the interaction site should be placed

    void `getParticleParameters`(int *particleIndex*, int &*parentIndex*, double &*sigma*, double &*epsilon*, double &*reductionFactor*) *const*¶
    :   Get the force field parameters for a vdw particle.

        |  |  |
        | --- | --- |
        | Parameters: |  |

        - **particleIndex** – the particle index
        - **parentIndex** – [out] the index of the parent particle
        - **sigma** – [out] vdw sigma
        - **epsilon** – [out] vdw epsilon
        - **reductionFactor** – [out] the fraction of the distance along the line from the parent particle to this particle at which the interaction site should be placed

    int `addParticle`(int *parentIndex*, double *sigma*, double *epsilon*, double *reductionFactor*)¶
    :   Add the force field parameters for a vdw particle.

        |  |  |
        | --- | --- |
        | Parameters: |  |

        - **parentIndex** – the index of the parent particle
        - **sigma** – vdw sigma
        - **epsilon** – vdw epsilon
        - **reductionFactor** – the fraction of the distance along the line from the parent particle to this particle at which the interaction site should be placed

        |  |  |
        | --- | --- |
        | Returns: | index of added particle |

    void `setSigmaCombiningRule`(*const* std::string &*sigmaCombiningRule*)¶
    :   Set sigma combining rule

        |  |  |
        | --- | --- |
        | Parameters: |  |

        - **sigmaCombiningRule** – sigma combining rule: ‘ARITHMETIC’, ‘GEOMETRIC’. ‘CUBIC-MEAN’

    *const* std::string &`getSigmaCombiningRule`(void) *const*¶
    :   Get sigma combining rule

        |  |  |
        | --- | --- |
        | Returns: | sigmaCombiningRule sigma combining rule: ‘ARITHMETIC’, ‘GEOMETRIC’. ‘CUBIC-MEAN’ |

    void `setEpsilonCombiningRule`(*const* std::string &*epsilonCombiningRule*)¶
    :   Set epsilon combining rule

        |  |  |
        | --- | --- |
        | Parameters: |  |

        - **epsilonCombiningRule** – epsilon combining rule: ‘ARITHMETIC’, ‘GEOMETRIC’. ‘HARMONIC’, ‘HHG’

    *const* std::string &`getEpsilonCombiningRule`(void) *const*¶
    :   Get epsilon combining rule

        |  |  |
        | --- | --- |
        | Returns: | epsilonCombiningRule epsilon combining rule: ‘ARITHMETIC’, ‘GEOMETRIC’. ‘HARMONIC’, ‘HHG’ |

    bool `getUseDispersionCorrection`() *const*¶
    :   Get whether to add a contribution to the energy that approximately represents the effect of VdW interactions beyond the cutoff distance. The energy depends on the volume of the periodic box, and is only applicable when periodic boundary conditions are used. When running simulations at constant pressure, adding this contribution can improve the quality of results.

    void `setUseDispersionCorrection`(bool *useCorrection*)¶
    :   Set whether to add a contribution to the energy that approximately represents the effect of VdW interactions beyond the cutoff distance. The energy depends on the volume of the periodic box, and is only applicable when periodic boundary conditions are used. When running simulations at constant pressure, adding this contribution can improve the quality of results.

    void `setParticleExclusions`(int *particleIndex*, *const* std::vector<int> &*exclusions*)¶
    :   Set exclusions for specified particle

        |  |  |
        | --- | --- |
        | Parameters: |  |

        - **particleIndex** – particle index
        - **exclusions** – vector of exclusions

    void `getParticleExclusions`(int *particleIndex*, std::vector<int> &*exclusions*) *const*¶
    :   Get exclusions for specified particle

        |  |  |
        | --- | --- |
        | Parameters: |  |

        - **particleIndex** – particle index
        - **exclusions** – [out] vector of exclusions

    void `setCutoff`(double *cutoff*)¶
    :   Set the cutoff distance.

    double `getCutoff`() *const*¶
    :   Get the cutoff distance.

    NonbondedMethod `getNonbondedMethod`() *const*¶
    :   Get the method used for handling long range nonbonded interactions.

    void `setNonbondedMethod`(NonbondedMethod *method*)¶
    :   Set the method used for handling long range nonbonded interactions.

    void `updateParametersInContext`(Context &*context*)¶
    :   Update the per-particle parameters in a `Context` to match those stored in this `Force` object. This method provides an efficient method to update certain parameters in an existing `Context` without needing to reinitialize it. Simply call `setParticleParameters()` to modify this object’s parameters, then call `updateParametersInContext()` to copy them over to the `Context`.

        The only information this method updates is the values of per-particle parameters. All other aspects of the `Force` (the nonbonded method, the cutoff distance, etc.) are unaffected and can only be changed by reinitializing the `Context`.

    bool `usesPeriodicBoundaryConditions`() *const*¶
    :   Returns whether or not this force makes use of periodic boundary conditions.

        |  |  |
        | --- | --- |
        | Returns: | true if nonbondedMethod uses PBC and false otherwise |

High performance molecular simulation on GPUs


### Navigation

- Core Objects
- Forces
- Integrators
- Extras

©2015, Stanford University and the Authors.
|
Powered by Sphinx 1.3.1
& Alabaster 0.7.7
|
Page source
